# Acquired SERPINC1/antithrombin deficiency during oral contraceptive consumption: a case report

**DOI:** 10.1186/s13256-023-04038-1

**Published:** 2023-07-28

**Authors:** D. Denora, M. V. Di Rosa, N. Altamura, F. Pellicori, P. Vinci, U. G. Sisto, F. Spanò, F. G. Di Girolamo, N. Fiotti, G. Biolo

**Affiliations:** 1 Department of Medical Surgical Ad Health Science, Clinica Medica, Cattinara Hospital, University of Trieste, Trieste, Italy; 2 SC Assistenza Farmaceutica, Cattinara Hospital, Azienda Sanitaria Universitaria Giuliano Isontina, Trieste, Italy; 3 SC Pronto Soccorso e Medicina d’urgenza, Cattinara Hospital, Azienda Sanitaria Universitaria Giuliano Isontina, Trieste, Italy

**Keywords:** Acquired SERPINC1, Oral contraceptives, Antithrombin deficiency, Thrombophilia, D-dimer, Case report

## Abstract

**Background:**

SERPINC1 is a glycoprotein that regulates blood coagulation. SERPINC1 congenital or acquired deficiencies represent a significant risk factor for thromboembolic disease. SERPINC1 acquired defects are observed in very few cases and can occur in many clinical conditions such as treatment with l-asparaginase or oral contraceptive (particularly estrogen derivatives), but these conditions are not routinely investigated.

**Case presentation:**

A 50-year-old Caucasian woman who took gestodene 75 µg/ethinylestradiol 20 µg as oral contraceptive, was sent to our thrombophilia clinic because, on thrombophilia testing, a reduction of SERPINC1 (74%) and a slight increase in circulating D-dimer and homocysteine were found. We investigated triggers of such SERPINC1 reduction, and identified gestodene 75 µg/ethinylestradiol 20 µg use as the most likely candidate. Two months after the discontinuation of the oral contraceptive, SERPINC1 value returned to normal (92%) and D-dimer and homocysteine were normalized.

**Conclusion:**

Each patient has a different sensitivity to contraceptive use. Genetic (or epigenetic) regulation of anticoagulant proteins might account for a different rate of consumption of anticoagulant proteins as oral contraceptives and probably determine the susceptibility to thrombotic events.

## Introduction

**Fig. 1:**
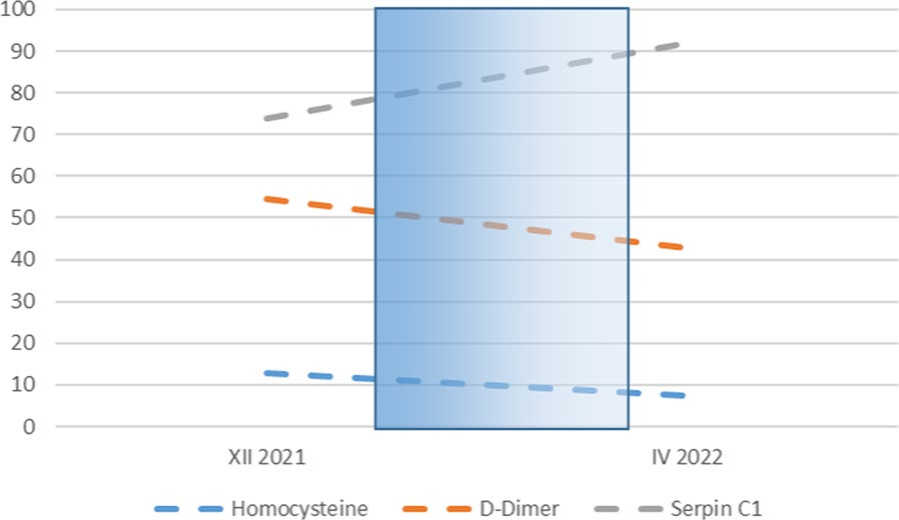
Time course of homocysteine, D-dimer, and SERPINC1 during observation

SERPINC1 (SR, also known as antithrombin) is a glycoprotein that regulates/inhibits blood coagulation and belongs to a group of inhibitory factors known as serpins (serine protease inhibitors) [1–3]. SR congenital or acquired deficiencies represent a significant risk factor for thromboembolic disease [4]. SR acquired defects (that is, lower activity) can occur in old age and can be triggered by liver cirrhosis or cancer, nephropathy, dyslipidemia, obesity, disseminated intravascular coagulation (DIC), sepsis, preeclampsia, treatment with l-asparaginase or oral contraceptive (in particular the estrogen derivatives), trauma, poisoning, and heparin therapy, but these conditions are not routinely investigated [1]. The SR reduction is observed in very few cases, and therefore, an individual susceptibility has to be postulated [5]. Some authors attributed such a reduction to estrogen-induced hemodilution [6]. In general, minor SR reductions (within 10%) [5, 6] can be observed and no treatment is needed for this finding, while the need for thromboprophylaxis in the above conditions has to be judged individually [7]. In the present case report, we describe a patient who developed an important acquired asymptomatic SR deficiency (−25%) following the use of oral contraceptives (Fig. 1).

## Case presentation

A 50-year-old housewife, Caucasian woman, nonsmoker, no history of alcohol or illicit drug abuse, has in her medical history mild systemic moderate hypertension (treated with bisoprolol 2.5 mg for many years), a history of herpes zoster virus trunk infection (shingles), gastroesophageal reflux disease, and hypercholesterolemia, and she had two full-term pregnancies She has been taking gestodene 75 µg/ethinylestradiol 20 µg (GE) as oral contraceptive since the age of 33.

In February 2022, the patient was referred to the emergency department because of recurrent episodes of heart palpitations. At admission, she had normal vital physiologic parameters (respiratory rate 20 breaths per minute, body temperature 36.8 °C, SpO_2_ 98%) and arterial blood pressure 170/90 mmHg, which normalized after 1.25 mg bromazepam per oral. Her body mass index was 29.9 kg/m^2^, and she had bilateral valgus knee; the rest of the physical examination was negative. Resting standard 12-lead electrocardiogram showed sinus rhythm (80 beats per minute) and normal repolarization. Her echocardiography was normal. Hypertension treatment was revised by a cardiologist who increased bisoprolol to 3.75 mg/day. She also reported having undergone thrombophilia testing in December 2021 because her daughter had two spontaneous abortions, and was suspected of carrying a genetic variant predisposing her to thrombophilia. The results of the testing were negative, both for the father and the daughter, while our patient showed SR activity at 74% (normal range 75–125%). The remaining coagulation test was normal: protein C resistance 0.94 (normal range 65–135 IU/dL), protein S activity 88%, antibodies for β2 glycoprotein and cardiolipin negative, lupus anticoagulants testing negative, and pathogenic variants of Factor V G1691A (rs6025), Factor V H1299R (rs1800595), PAI1 5G/5G (rs1799889), FII G20210A (rs1799963), and MTHFR C677T (rs1801133) polymorphisms negative. The reasons for referral to our center were for clinical and laboratory workup of the low SR level, and of simultaneous slightly elevated D-dimer (632 mg/L, normal value < 500 mg/L) and homocysteine 12.7 μmol/L (normal range 5–12 μmol/L) plasma levels.

After collecting the patient’s personal history, and investigating possible triggers of such SR reduction, oral contraceptive (OC) use remained the most likely candidate for this modification [1, 7]. Since a final confirmation of such a role could only come from suspension, we asked to stop OC for at least 30 days and avoid any drug potentially interfering with blood coagulation. The patient initially accepted to temporarily suspend OC (at the end of February 2022) and made a definitive change In April 2022. By the end of April, the SR activity, D-dimer, and homocysteine plasma levels were back to normal: SR rose from 74% to 92%, D-dimer decreased from 632 to 427 mg/L, and homocysteine went from 12.7 to 7.4 μmol/L. At the last follow-up visit (October 2022) the patient was in good clinical condition.

## Discussion and conclusion

Oral contraceptives are known to trigger intimal proliferation in venous vessels, increase venous distensibility, and therefore decrease venous flow, thus accounting for thrombus risks [8–10]. These drugs are also able to boost blood coagulability by increasing fibrinogen, factor VII, VIII, IX, X, and XII plasma levels, by shortening platelet aggregation time, and by decreasing SR activity [11]. It is known that the risk of developing a thrombus while assuming GE is between 9 and 12 women per 10,000 per year [12]. In studies evaluating the safety profile, the lowest reported SR activity value during GE consumption was well above 90% [13, 14]. The low SR activity (74%) observed during GE consumption in our patients was remarkable for two reasons: the first is that it is almost isolated, and the second is that it is quite lower than those previously published [6, 11, 15]. In our experience, patients with SR activity lower than 80% can sometimes have consumption coagulopathy, and, even if negative for this condition, a reason needs to be identified. An alternative explanation for such a selective reduction of only one marker, as observed in our patient, was a mild or well-compensated SR genetic defect. In this context, the decision to stop hormonal contraception has been critical to identify this condition. The complete recovery observed within a few weeks from suspension with a 20% gain in activity opens a scenario of a selective consumption of inhibition of SR played by GE. Literature reports very few cases of vascular events due to isolated SR deficiency (with activity ranging from 9% to 60%) combined with oral contraception [2, 16], but only one without vascular events [17], while no case reports have demonstrated an acquired SR deficiency induced by combination of gestodene and ethinylestradiol. To the best of our knowledge, this is also the first case without events and without familial SR deficiency. This raises the speculation that each subject might have a different responsiveness to contraceptive use, even if the time course in this patient was event free. The current observation suggests that a genetic (or epigenetic) regulation of anticoagulant proteins might account for a different rate of consumption of anticoagulant proteins during OC (or, for example, pregnancy or infection). Furthermore, a strategy to screen women who have an atypical reaction to GE (or other OC) is missing, and this tool might help to prevent complications of oral contraception in the future. Possible ways could be the combination of *in vivo* coagulation test (D-dimer, but also F1 + 2, thrombin–antithrombin complexes, and fibrinopeptide A) with clinical variables or, given the plummeting costs of this technology, genomic analysis. In conclusion, our observation might shed some light on the complex scenario of the individual sensitivity to contraceptives and suggest that further effort is required to make these drugs safer.

## Data Availability

Clinical data are not publicly available, but they can be obtained from the Head of the Unit Clinica Medica.

## Abbreviations

SR: SERPINC1 (antithrombin)
GE: Gestodene 75 µg/ethinylestradiol 20 µg
OC: Oral contraceptives
